# A comprehensive dosimetric study of Monte Carlo and pencil‐beam algorithms on intensity‐modulated proton therapy for breast cancer

**DOI:** 10.1002/acm2.12497

**Published:** 2018-11-28

**Authors:** Xiaoying Liang, Zuofeng Li, Dandan Zheng, Julie A. Bradley, Michael Rutenberg, Nancy Mendenhall

**Affiliations:** ^1^ Department of Radiation Oncology University of Florida College of Medicine Gainesville FL USA; ^2^ Department of Radiation Oncology University of Nebraska Medical Center Omaha NE USA

**Keywords:** breast cancer, dose algorithms, IMPT

## Abstract

PB algorithms are commonly used for proton therapy. Previously reported limitations of the PB algorithm for proton therapy are mainly focused on high‐density gradients and small‐field dosimetry, the effect of PB algorithms on intensity‐modulated proton therapy (IMPT) for breast cancer has yet to be illuminated. In this study, we examined 20 patients with breast cancer and systematically investigated the dosimetric impact of MC and PB algorithms on IMPT. Four plans were generated for each patient: (a) a *PB plan* that optimized and computed the final dose using a PB algorithm; (b) a *MC‐recomputed plan* that recomputed the final dose of the PB plan using a MC algorithm; (c) a *MC‐renormalized plan* that renormalized the MC‐recomputed plan to restore the target coverage; and (d) a *MC‐optimized plan* that optimized and computed the final dose using a MC algorithm. The DVH on CTVs and on organ‐at‐risks (OARs) from each plan were studied. The Mann–Whitney *U*‐test was used for testing the differences between any two types of plans. We found that PB algorithms significantly overestimated the target dose in breast IMPT plans. The median value of the CTV D_99%_, D_95%_, and D_mean_ dropped by 3.7%, 3.4%, and 2.1%, respectively, of the prescription dose in the MC‐recomputed plans compared with the PB plans. The magnitude of the target dose overestimation by the PB algorithm was higher for the breast CTV than for the chest wall CTV. In the MC‐renormalized plans, the target dose coverage was comparable with the original PB plans, but renormalization led to a significant increase in target hot spots as well as skin dose. The MC‐optimized plans led to sufficient target dose coverage, acceptable target hot spots, and good sparing of skin and other OARs. Utilizing the MC algorithm for both plan optimization and final dose computation in breast IMPT treatment planning is therefore desirable.

## 1 INTRODUCTION

Due to its unique energy absorption profile, proton therapy has several critical advantages over photon therapy. It provides excellent target coverage and minimized cardiac and pulmonary exposure for post‐lumpectomy and post‐mastectomy irradiation.^1^, ^2^, ^3^ Indeed, the interest in proton therapy for breast cancer has substantially increased over the past decades, as evidenced by the recently opened, 1700‐patient, randomized trial of proton vs photon therapy for breast cancer patients (RADCOMP, NCT: 02603341)^4^ and the many publications on this subject.^5^

Clinical dose calculations for proton therapy are primarily obtained using the pencil‐beam (PB) algorithm, which assumes that the material on the central axis is laterally infinite and the modeling of nuclear reaction and multiple Coulomb scattering can only be approximate. This leads to inaccurate dose distributions in the presence of complex geometries and heterogeneous environments.^6^ In comparison, the Monte Carlo (MC) algorithm simulates particle propagation through materials by randomly sampling the cross section of interactions.^7^ Thus, MC dose calculation is considered the most accurate method to compute doses in radiation therapy. Several phantom studies comparing proton dose distributions calculated with PB and MC algorithms have demonstrated that the MC algorithm provides more accurate treatment planning than the PB algorithm.^8^, ^9^, ^10^, ^11^, ^12^ For example, the recently published Imaging and Radiation Oncology Core (IROC) lung phantom study^12^ demonstrated that the dose distribution obtained with the MC algorithm, as compared with the PB algorithm, more closely matched the phantom dose measurement. However, in spite of the reported limitations of the PB algorithm, it is still regarded as the standard of care in proton therapy due to the following: (a) it allows for fast treatment planning, leading to more efficient clinical workflow; and (b) the relative scarcity of dosimetric results on actual patients showing the resulting dose errors. Most of the existing studies evaluating algorithms were conducted on phantoms and focused on high‐density gradients and small‐field dosimetry. Although a limited number of studies^13^, ^14^, ^15^ have evaluated the dose calculation errors in patients, the primary focus was still high‐gradient tissue inhomogeneity. Due to the high‐gradient tissue inhomogeneity involved in treatment sites such as the thorax, large PB accuracy deficiencies are expected and have been confirmed by previous studies.^14^, ^15^ However, sites such as the breast and chest wall (CW), have not been thoroughly investigated. The accuracy of the PB algorithm may not initially be questioned for the more homogenous site of breast. However, due to the use of a range shifter and the presence of a relatively large air gap in intensity‐modulated proton therapy (IMPT) for breast cancer, PB algorithms may lead to meaningful dose distribution calculation errors. Indeed, in the benchmark study of RayStation (RaySearch Laboratories, Stockholm, Sweden) PB and MC dose calculation algorithms, Saini et al.^10^ found that dose discrepancies of up to 8% occurred at shallow depths between the phantom measurement and the PB computation for a large air gap situation when a range shifter was used. Proton therapy for breast cancer falls into this same category, namely the combination of the use of a range shifter and a relatively large air gap between the range shifter and the patient. Clearly, studies of the dose errors inherent in the PB algorithm for IMPT breast cancer cases are necessary.

In the current study, we systematically investigated the dosimetric impact of MC and PB algorithms on IMPT planning optimization and final dose computation for breast cancer treatment. Four plans were generated for each patient: (a) a *PB plan* that optimized and computed the final dose using a PB algorithm; (b) a *MC‐recomputed plan* that recomputed the final dose of the PB plan using a MC algorithm; (c) a *MC‐renormalized plan* that renormalized the MC‐recomputed plan to restore the target coverage; and (d) a *MC‐optimized plan* that optimized and computed the final dose using a MC algorithm. The aims of the study were as follows: (a) to quantify the dose errors produced by the PB algorithm by comparing the PB plans and the MC‐recomputed plans; and (b) by comparing the MC‐renormalized plan against the MC‐optimized plan, to evaluate if MC‐renormalized plan offers a good balance between dose accuracy and planning efficiency. To the best of our knowledge, this is the first comprehensive study of MC and PB algorithms in IMPT for breast cancer.

## 2 MATERIALS AND METHODS

The study was approved by the Institutional Review Board.

### 2.A Patients

This single institution study consisted of 20 female patients (8 post‐mastectomy and 12 post‐lumpectomy) who received IMPT to the whole breast or CW and regional lymph nodes (with the exception of one patient who received IMPT to the breast only) between 06/2017 and 06/2018. Patients received IMPT to the breast/CW and internal mammary nodes (IMN), axillary level I–III nodes (AxI‐III), and supraclavicular nodes (SCV) (*n *= 17); breast and IMN and AxI‐III (*n* = 1); CW and IMN (*n* = 1); and breast only (*n* = 1).

### 2.B Simulation, target volumes, and OARs

All patients were simulated in the supine position with arms above their heads using a standard wing board and a Vac‐Lok immobilization bag. Four‐dimensional computed tomography (4DCT) scans with a slice thickness of 2 mm were acquired using a Philips Brilliance Big Bore CT (Philips Healthcare, Eindhoven, The Netherlands). The average CT images were transferred to MIM (MIM Software Inc., Beachwood, OH) for contouring. The CTV structures, including breast tissue or CW, IMN, AxI, AxII, AxIII, and SCV, were contoured on the average CT images. All CTV structures were combined to generate the total CTV, which was expanded by 5 mm (excluding the skin, heart, esophagus, thyroid, and lung +3 mm), and then smoothed to create the planning PTV. Organ‐at‐risk (OAR) structures, including the heart, ventricles (combined right and left), left anterior descending artery (LAD), left lung, right lung, esophagus, and thyroid, were also contoured on the average CT. A layer skin structure of 5 mm (for intact breasts) or 3 mm (for CW) inward from the body was also contoured.

### 2.C Treatment planning

The average CT images and the contours were transferred to RayStation (V6.1) for treatment planning. The dose prescription was 50 Gy(RBE) in 25 fractions. For each plan, two en‐face angles between 0° and 30° were used. A water equivalent 7.4‐cm Lucite ranger shifter was used for each beam. The selective robust optimization strategy^16^ with both robust objectives and normal objectives was used to achieve a robust plan against uncertainties with desirable dose distribution. To be more detailed, the plan was robust optimized on all CTV structures with 5‐mm setup uncertainty and 3.5% range uncertainty, and normal optimization on the planning PTV was also included in the objective function. The OARs were optimized on the nominal scenario as they receive minimum dose and no risk of exceeding the tolerance under setup and range error scenarios. All optimizations were carried out on a 2‐mm calculation grid for 200 iterations. The target dose was evaluated on CTVs, as detailed below. For each patient, four plans were generated using RayStation TPS:

1. *PB plan:* The PB algorithm was used for optimization and final dose computation. The plan was normalized to 95% of the PTV covered by 95% of the prescription dose.
2. *MC‐recomputed plan:* The PB plan was recomputed using the MC algorithm with 0.5% statistical uncertainty. The statistical error is the mean one standard deviation error over all voxels having a dose above 50% of the maximum dose. Identical pencil‐beam scanning (PBS) energy layers, spot geometry and weighting, and monitor units were used for the recomputation.
3. *MC‐renormalized plan:* The MC‐recomputed plan was renormalized to 95% of the PTV covered by 95% of the prescription dose.
4. *MC‐optimized plan:* While maintaining the identical objective function, including the robust optimization settings used in the PB plan, but resetting the PBS energy layers, spot geometry and weighting and monitor units, the plan was then reoptimized using the MC algorithm with a sampling history of 50,000 ions/spot, and a final dose computed using the MC algorithm with 0.5% statistical uncertainty. Thereafter, the plan was normalized to 95% of the PTV covered by 95% of the prescription dose.

### 2.D Dosimetric evaluation and comparison

For all of the above plans, the hot spot dose received by 2% of the volume (D_2%_), the minimum dose received by 99% of the volume (D_99%_), the mean dose (D_mean_), the relative volume that received 95% of the prescription dose (V_95%_), and the dose received by 95% of the total CTV and each CTV (CTV breast/CW, CTV IMN, CTV AxI, CTV AxII, CTV AxIII, CTV SCV) (D_95%_) were recorded. The treatment goal was for 95% of each CTV to receive at least 95% of the prescription dose. The dose volume information for OARs was studied for the heart, ventricles, LAD, ipsilateral lung, contralateral lung, esophagus, thyroid, and skin. In addition, the Radiation Therapy Oncology Group (RTOG) conformity index (CI) and homogeneity index (HI) were calculated. The CI was defined as $\left(V_{95\%}^{\mathrm{Target}}/V_{95\%}^{\mathrm{Body}}\right)$, where $V_{95\%}^{\mathrm{Target}}$ is the target volume receiving 95% of the prescription dose and $V_{95\%}^{\mathrm{Body}}$ is the total irritated volume receiving 95% of the prescription dose. Here, we used 95% of the prescription dose in the calculation instead of 100% because all plans were normalized to D_95%_ to 95% of the prescription dose. The HI was defined as the ratio of D_95%_ over D_5%_. For any plan, the CI and HI fell in the range of 0 to 1.0, with CI = 1.0 for an ideally conformal plan and HI = 1.0 for an ideally homogeneous plan. The Mann–Whitney *U*‐test was used for testing the differences between any two types of plans. A *P *< 0.05 was considered to be statistically significant.

## 3 RESULTS

**Figure 1:**
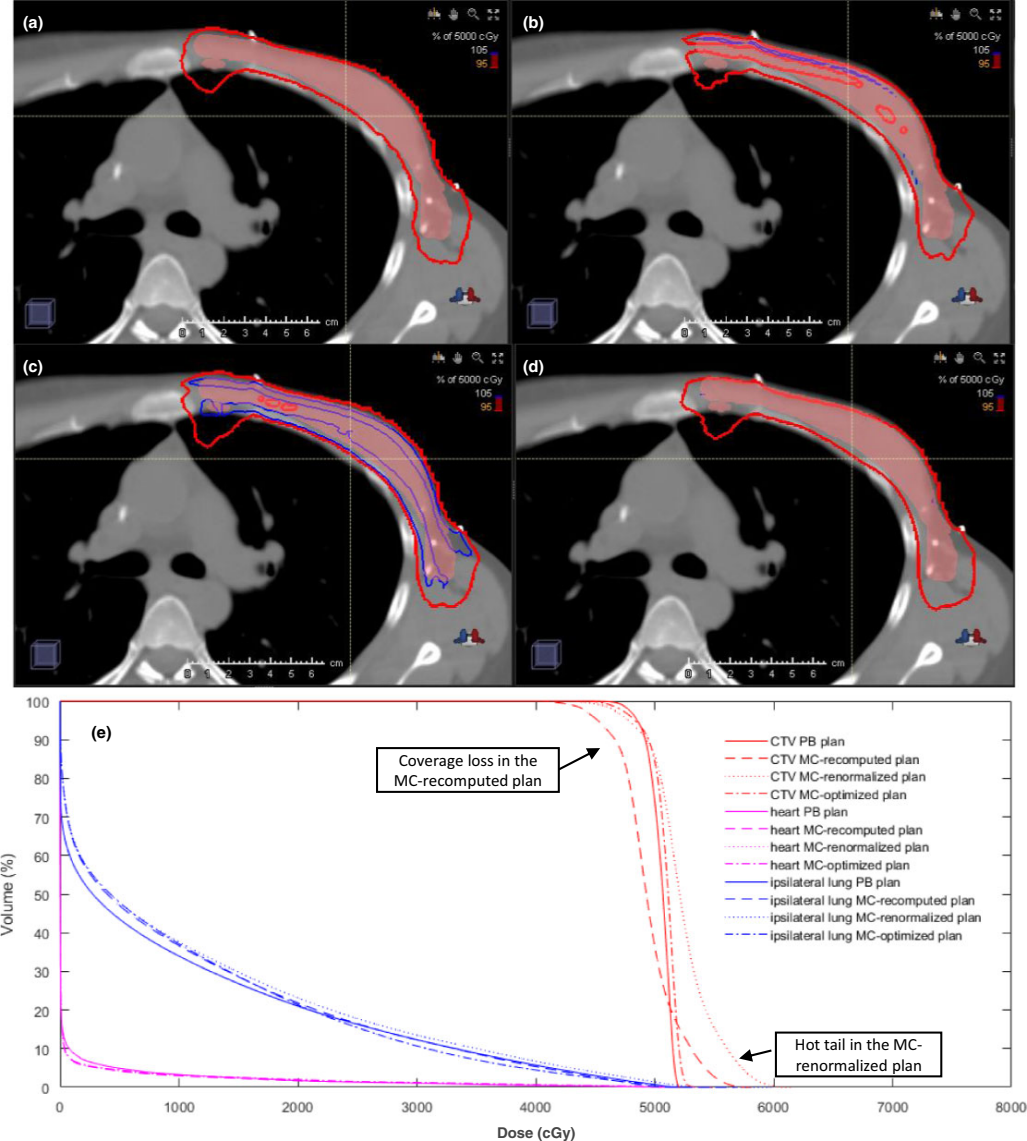
Dose distributions on an example patient: (a) the PB plan, (b) the MC‐recomputed plan, (c) the MC‐renormalized plan, and (d) the MC‐optimized plan. To ensure legibility, only the 95% (red) and 105% (blue) isodoses are shown. The color‐washed structure is the total CTV. A DVH plot superimposing all four plans is shown in (e).

Figure 1 illustrates the isodose distributions of an example patient planned with (a) the PB plan, (b) the MC‐recomputed plan, (c) the MC‐renormalized plan, and (d) the MC‐optimized plan. Figure 1(e) illustrates a dose‐volume histogram (DVH) plot superimposing all four plans. The MC‐recomputed plan led to substantially reduced CTV coverage, as compared with the PB plan. The PB plan also artificially overestimated the dose distribution uniformity in the CTV. While the MC‐renormalized plan restored the CTV D_95%_ coverage, it had a much larger tail in the high‐dose region on the DVH plot compared with the PB plan. The MC‐optimized plan provided optimal CTV coverage and homogeneous dose distribution.

Table 1 lists the dose statistics for the total CTV in the PB plans, the MC‐recomputed plans, the MC‐renormalized plans, and the MC‐optimized plans. When the PB plans were recomputed using MC for the final dose, the median value of CTV D_99%_, D_95%_, and D_mean_ dropped by 3.7%, 3.4%, and 2.1%, respectively, of the prescription dose, which rendered the plans not meeting our treatment goal of CTV D_95%_ ≥ 95% of the prescription dose. The median value of CTV volume that receives 95% of the prescription dose (CTV V_95%_) dropped by 21.8%. The CTV dose coverage reductions were all statistically significant, with *P* < 10^−5^. The mean and standard deviation (SD) of the dose difference between the MC‐recomputed plans and PB plans on CTV D_99%_, D_95%_, and D_mean_ were 3.3% (2.1%), 3.5% (1.0%), and 2.0% (0.4%) of the prescription dose, respectively. The mean and SD of the CTV V_95%_ difference was 19.8% (10.2%). After renormalization (the MC‐renormalized plans), the CTV D_95%_, and D_99%_ were restored (Table 1), essentially comparable to the original PB plans, with *P*‐values of 0.24 and 0.18, respectively. However, compared with the PB plans, the DVH in the MC‐renormalized plan showed a much more gradual dose fall off in the CTV with a much longer “hot” tail [Fig. 1(e)]. As a result, the HI (D_95%_/D_5%_) was reduced from 0.94 (0.93–0.95) in the PB plans to 0.92 (0.85–0.94) in the MC‐renormalized plans with a *P* < 10^−6^. Nevertheless, the CTV dose coverage was satisfactorily recovered in the MC‐renormalized plans as per our treatment goal, although with increased target dose heterogeneity. In contrast, the MC‐optimized plans offered optimal CTV coverage and homogeneous dose distribution, achieving dose distributions similar to the PB plan, but accurately calculated by the MC algorithm.

**Table 1:** Median (range) of CTV doses and relative volumes that receive 95% of the prescription dose in four types of plans

Dosimetric parameters	PB plans	MC‐recomputed plans	MC‐renormalized plans	MC‐optimized plans	*P*‐values PB vs MC‐recomputed plans	*P*‐values PB vs MC‐renormalized plans	*P*‐values MC‐renormalized vs MC‐optimized plans
CTV D_99%_	94.2% (82.0%–95.2%)	90.5% (85.2%–92.0%)	93.8% (87.8%–94.8%)	93.1% (82.8%–95.6%)	1.16E‐06	0.18	0.31
CTV D_95%_	96.1% (94.8%–99.4%)	92.7% (90.4%–96.2%)	95.9% (94.6%–99.0%)	96.2% (95.0%–99.8%)	4.07E‐07	0.24	0.07
CTV V_95%_	98.2% (94.2%–99.4%)	76.4% (54.9%–97.5%)	97.6% (93.0%–98.8%)	98.0% (94.9%–99.7%)	1.42E‐07	0.04	0.34
CTV D_mean_	98.8% (98.2%–102.0%)	96.7% (95.4%–100.6%)	100.2% (98.6%–104.6%)	99.2% (97.8%–103.4%)	4.54E‐06	1.45E‐04	0.006
CTV D_2%_	101.5% (100.4%–105.4%)	102.3% (100.0%–111.6%)	106.2% (102.4%–117.0%)	102.0% (100%–115.8%)	0.05	4.69E‐07	8.52E‐06
HI	0.94 (0.93–0.95)	0.92 (0.85–0.94)	0.92 (0.85–0.94)	0.94 (0.92–0.96)	5.81E‐07	5.81E‐07	1.57E‐05
CI	0.85 (0.61–0.94)	0.88^a^ (0.65–0.95)	0.82 (0.56–0.92)	0.86 (0.61–0.93)	0.23	0.34	0.19

The doses are displayed as percentage of the prescription dose.

a The CI on the MC‐recomputed plans is not a good plan quality evaluation metric as the CTV coverage is compromised.

In the studied patient cohort, there are three patients with fewer targets of lymph nodes. We have reviewed the data on these three patients and found that there is no systematical trend in these three patient's data falling close to the either extreme of the range on any studied dose indices. Therefore, including these three cases does not introduce bias in the results.

### 3.A Impact of final dose computation algorithm on each CTV structure:

**Figure 2:**
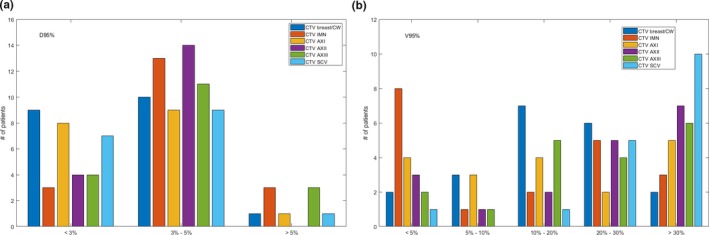
Patient distribution of the dose overestimation magnitude on (a) D_95%_ and (b) V_95%_ of each CTVs by the PB plans when compared with MC‐recomputed plans. The total number of patients treated to CTV breast/CW, CTV IMN, CTV AXI‐III, and CTV SCV are 20, 19, 18, and 17, respectively.

As shown above, the PB algorithm overestimated the target coverage. We further analyzed the target coverage in terms of D_95%_ and V_95%_ for each CTV structure separately (Table 2). Figures 2(a) and 2(b) plot the distribution of the dose overestimation magnitude on D_95%_ and V_95%_ for individual CTVs in the PB plans, as compared with the MC‐recomputed plans. The percentage of patients with more than 3% of D_95%_ overestimation in the PB plans were: 55% (11/20) on CTV breast/CW, 84% (16/19) on CTV IMN, 56% (10/18) on CTV AxI, 78% (14/18) on CTV AxII and CTV AxIII, and 59% (10/17) on CTV SCV. The percentage of patients with more than 20% of V_95%_ overestimation were: 40% (8/20) on CTV breast/CW, 50% (8/19) on CTV IMN, 39% (7/18) on CTV AxI, 67% (12/18) on CTV AxII, 56% (10/18) on CTV AxIII, and 88% (15/17) on CTV SCV.

**Table 2:** Median (range) of D_95%_ and V_95%_ overestimation on each CTV structure by the PB plans compared with the MC‐recomputed plans. The Dose differences (PB plans — MC‐recomputed plans) on OARs also shown here

CTVs	Breast/CW	IMN	AXI	AXII	AXIII	SCV
ΔD_95%_	3.0% (2.0%–7.4%)	3.7% (1.0%–8.8%)	3.0% (2.2%–5.2%)	3.2% (2.6–4.2%)	3.4% (2.6–5.2%)	4.8% (3.8–6.8%)
ΔV_95%_	19.6% (1.8%–40.7%)	13.0% (0.1%–33.4%	15.5% (2.4%–60.1%)	24.9% (0.8%–50.1%)	25.0% (0.5%–59.3%)	39.0% (1.1%–59.9%)
OARs	Heart ΔD_mean_ (Gy)	Ipsi‐lung ΔV_20Gy_ (%)	Esophagus ΔD_max_ (Gy)	Thyroid ΔD_mean_ (Gy)		
	0 (−0.1 to 0.2)	−1.4 (−2.8 to 0)	−0.1 (−1.4 to 1.4)	0.8 (−0.7 to 1.5)		

**Figure 3:**
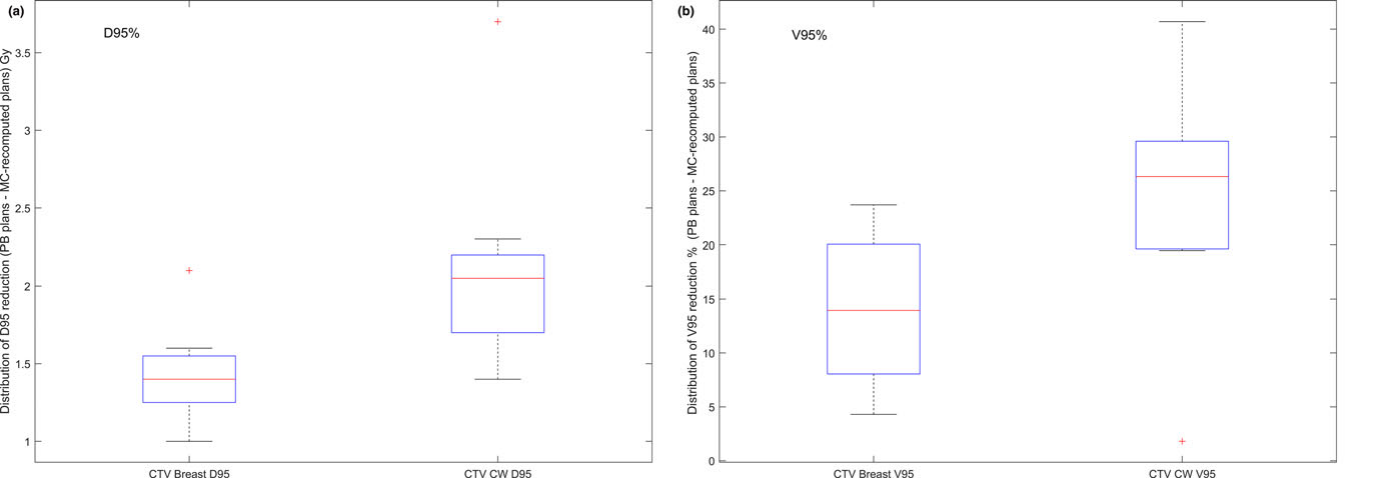
Box plot of (a) D_95%_ and (b) V_95%_ overestimation on CTV breast and on CTV CW by the PB plans when compared with MC‐recomputed plans.

We further analyzed the data by separating CTV breast and CTV CW. The median (range) of the D_95%_ reductions on CTV breast and CTV CW were 1.4 (1.0–2.1) Gy and 2.0 (1.4–3.7) Gy, corresponding to 2.8% (2%–4.2%) and 4.0% (2.8%–7.4%) of the prescription dose, respectively. The median (range) of the V_95%_ reductions on CTV breast and CTV CW were 14.0% (4.3%–23.7%) and 24.0% (1.8%–40.7%), respectively. The differences between CTV breast and CTV CW on the V_95%_ and D_95%_ reductions were statistically significant, with *P* < 10^−4^ and 0.004, respectively. Figures 3(a) and 3(b) show the box plot of V_95%_ and D_95%_ reductions of the CTV breast and CTV CW in the MC‐recomputed plans, as compared with the PB plans. The CTV CW had a higher likelihood of larger V_95%_ and D_95%_ overestimation in the PB plans.

Table 2 also lists the dose differences on the OARs between the PB plans and the MC‐recomputed plans. The dose differences on the heart and lung were small [as also observed in Fig. 1(e)] and there was no obvious trend in over‐ or under‐estimation of the PB algorithm computed dose. This is likely due to two reasons: (a) the doses to the heart and the lung are minimum, and (b) the effect of inaccurate modeling of scatter in the range shifter and the air gap by the PB algorithm is more problematic at shallow depths. In our case, since en‐face beams were used, the OARs such as heart and lung are in the downstream to the CTV and therefore much less affected by the modeling inaccuracies due to the range shifter and the air gap. That is in agreement with the findings of the phantom study by Saini et al.^10^ that large dose discrepancy was found at shallower depths.

### 3.B Comparison between MC‐renormalized plans and MC‐optimized plans

As shown above, the PB plans significantly overestimated the dose to target, and the MC‐renormalized plans were able to restore the target coverage. Nonetheless, the target dose heterogeneity increased upon MC‐renormalization and exceeded acceptable levels in some cases. We, therefore, investigated the effect of MC optimization. Both the MC‐renormalized plans and MC‐optimized plans met our CTV coverage goal of D_95%_ ≥ 95% of the prescription dose. However, the MC‐renormalized plans had a significantly higher target hot‐spot dose (D_2%_) than the MC‐optimized plans.

Since the MC‐renormalized plans and MC‐optimized plans were able to meet the treatment goal on the CTVs, we compared the dose volume information on OARs to see which plan provided better sparing. Table 3 summarizes the median (range) of the dose volume information on OARs. The doses to the heart, ventricles, and LAD are very small for the right breast/CW patients. Therefore, only the data from the left breast/CW patients were included in the study of these OARs. Similarly, only the data from patients who received the SCV irradiation were included for the study of esophagus and thyroid. As Table 3 shows, the MC‐renormalized plans and MC‐optimized plans led to similar dose to the heart, ventricles, lungs, and thyroid, but the MC‐optimized plans reduced the maximum dose to the esophagus. The MC‐optimized plans were also able to provide significantly better skin sparing than the MC‐renormalized plans. The MC‐optimized plans showed better CI and HI compared with the MC‐renormalized plans (Table 3), although the improvement in CI was not statistically significant. Consistent with significantly higher hot spots (D_2%_) in the MC‐renormalized plans, the HI was significantly improved (closer to 1) in the MC‐optimized plans than in the MC‐renormalized plans.

**Table 3:** Median(range) of the dose volume information on the OARs. The plan quality indices of CI and HI are also shown

	Heart				
	V_5Gy_ (%)	V_10Gy_ (%)	V_25Gy_ (%)	D_5%_ (Gy)	Mean dose (Gy)
MC‐renormalized	4.9 (1.1–8.4)	3.0 (0.2–6.0)	1.3 (0–3.0)	4.7 (0.9–13.7)	1.1 (0.2–2.2)
MC‐optimized plan	4.7 (0.7–8.7)	3.0 (0–6.1)	1.1 (0–3.0)	4.6 (1.1–14.0)	1.1 (0.2–2.2)
*P*‐value	0.82	0.84	0.95	0.9	0.9

	Ventricles		LAD		
	V_5Gy_ (%)	V_25Gy_ (%)	Mean dose (Gy)	D_0.1cc_ (Gy)	
MC‐renormalized	4.7 (0.1–7.2)	0.8 (0–2.2)	3.4 (0.3–11.5)	13.1 (1.2–33.3)	
MC‐optimized plan	3.7 (0–6.8)	0.7 (0–1.9)	3.5 (0.4–10.8)	13.2 (1.1–30.2)	
*P*‐value	0.77	1	1	0.68	

	Ipsilateral‐lung			Contralateral‐lung	
	V_5Gy_ (%)	V_10Gy_ (%)	V_20Gy_ (%)	V_5Gy_ (%)	
MC‐renormalized	44.4 (30.7–62.9)	31.8 (17.5–51.2)	17.3 (6.6–32.9)	1.7 (0–9.5)	
MC‐optimized plan	43.3 (30–61.8)	30.3 (16.9–49.3)	16.3 (6.2–30.3)	1.9 (0–9.6)	
*P*‐value	0.72	0.68	0.54	0.92	

	Esophagus		Thyroid		
	Mean dose (Gy)	Max dose (Gy)	Mean dose (Gy)		
MC‐renormalized	11.9 (5.3–31.6)	49.5 (43.2–52.2)	26.4 (18.8–32.1)		
MC‐optimized plan	11.8 (5.3–31.9)	48.4 (44.3–50.5)	26.7 (18.9–32.6)		
*P*‐value	1	0.005	0.84		

	Skin			Plan quality indices	
	Max dose (Gy)	D_10cc_ (Gy)	V_52.5Gy_ (cm^3^)	CI	HI
MC‐renormalized	57.0 (51.4–80.6)	51.2 (47.4–65.9)	4.0 (0–55.0)	0.82 (0.56–0.92)	0.92 (0.85–0.94)
MC‐optimized plan	52.2 (50.2–77.5)	49.3 (47.1–64.1)	0 (0–35.4)	0.86 (0.61–0.93)	0.94 (0.92–0.96)
*P*‐value	9.75E‐05	0.002	3.31E‐04	0.19	1.57E‐05

**Figure 4:**
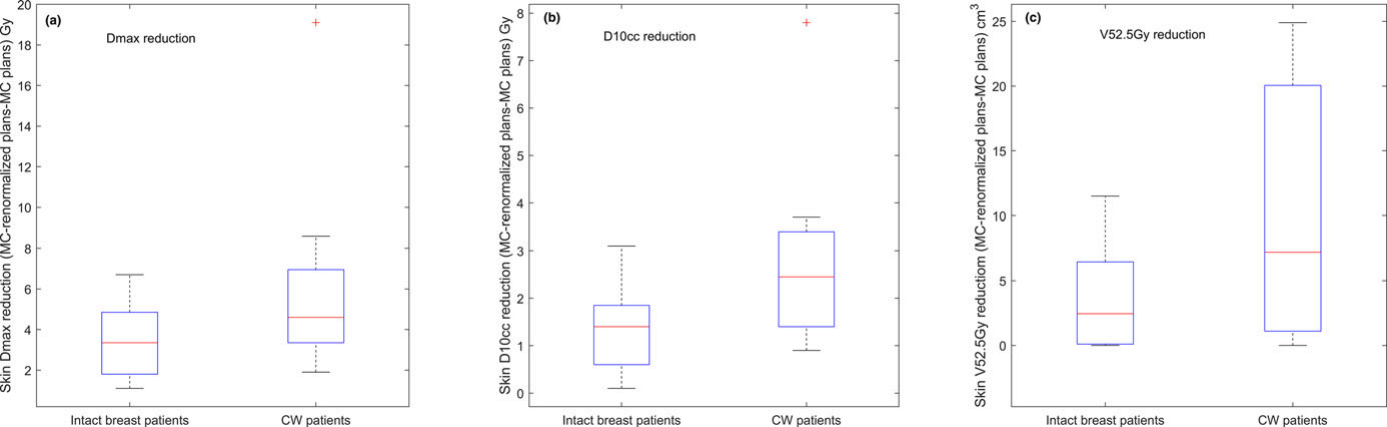
Distribution of skin sparing improvement on (a) Dmax, (b) D10cc, and (c) V52.5Gy for the intact breast group and CW group in the MC plans compared with the MC‐renormalized plans.

There were no appreciable differences between the MC‐renormalized and MC‐optimized plans when post‐lumpectomy vs post‐mastectomy patients were compared for dose to the heart, ventricles, lungs, esophagus, and thyroid. However, skin sparing in the MC‐optimized plans compared with the MC‐renormalized plans showed a greater improvement in the CW patients than in the intact breast patients, although this was not statistically significant (*P *= 0.20, 0.07, and 0.20 on skin max dose, skin D_10cc_, and skin V_52.5Gy_, respectively). Figure 4 shows the distribution of skin sparing improvement for intact breast patients and CW patients in the MC‐optimized plans compared with the MC‐renormalized plans.

## 4 DISCUSSION

Most published studies detailing the weaknesses of the PB algorithm in proton therapy have been conducted on phantoms. It is important to translate the improved accuracy with MC dose computation to clinical patient cases and determine the expected differences in dose distributions using the MC algorithm. To date, few studies have investigated the dose calculation errors in clinical patient cases.^13^, ^14^, ^15^ In a study of 10 patients for lung proton PBS treatment, Maes et al. observed a median decrease of 10% on CTV V_95%_ when the PB plans were recomputed with the MC algorithm^14^ In a recent seminal study, Yepes et al. analyzed MC and PB dose computations for IMPT plans on 525 patients (including four treatment sites — the brain, head and neck, thorax, and prostate) and found an up to 10% target dose difference for 2% of the thoracic patients.^15^ In the only published study assessing the impact of analytical dose calculation algorithms on breast treatment, Schuemann et al. found an up to 4% D_95%_ error among 10 breast cancer patients treated with double‐scattered proton therapy, when compared with the MC algorithm.^13^ However, this dose error may not directly translate to PBS treatment for breast cancer, due to the different components that the proton beam passes through before reaching the patient between these two delivery techniques.

We conducted a comprehensive study in 20 clinical breast cancer cases treated with IMPT to investigate the impact of dose algorithms on treatment plans. Our main concern when analyzing the dose difference in the target volume was whether the PB algorithm would substantially overestimate the target dose as compared with the MC algorithm. Thus, if the PB algorithm was used for planning, the target would be irradiated at a lower dose than prescribed, thereby potentially increasing the risk of recurrence. We found that the PB plans significantly overestimated the dose to the target. These dose overestimations by the PB algorithm warrant further investigation to assess clinical significance.

Although the MC algorithm models the beam more accurately than the PB algorithm, the extended optimization time, especially when robust optimization is applied, is a major concern for planning efficiency. We have found that an IMPT plan robust optimized on 147 scenarios with a MC algorithm for a breast and regional lymph nodes case on average takes ~20 hr for 200 iterations with 50,000 ions/spot; however, the same case optimized with the PB algorithm only requires approximately ¼ of the time. To achieve an optimal clinical treatment planning workflow, both accurate dose calculation and computation efficiency are critical. Therefore, it is natural to ask the question if the MC‐renormalized plan, which takes advantage of the faster PB optimization and the more accurate MC final dose computation, could be an ideal choice for treatment planning. Thus, we also evaluated the MC‐renormalized plans against MC‐optimized plans. The results showed that MC‐renormalized plans restored the prescribed dose to the target; however, they also led to a significant increase in hot spots, as compared with the MC‐optimized plans. In addition, the MC‐renormalized plans suffered from significantly worse skin sparing compared with the MC‐optimized plans; this may be attributable to that our objective function included a maximum skin dose constraint, since radiation dermatitis probability is correlated with the maximum dose and hot spot. The MC‐renormalized plans were optimized using the PB algorithm; the PB algorithm was not able to predict the dose accurately enough during optimization, translating to sub‐optimal dose distribution when the final dose was recomputed and renormalized using the MC algorithm. In comparison, the MC‐optimized plans were more accurately calculated during the optimization to achieve the objectives. Radiation dermatitis is a well‐recognized risk of proton therapy for breast cancer.^17^, ^18^ Increased skin toxicity has long been considered a potential limiting factor in the clinical use of protons for breast cancer, although proton therapy offers significant advantages of minimizing cardiac and pulmonary dose. A recent study of prognostic factors of grade 3 radiation dermatitis in breast patients treated with proton therapy demonstrated that skin hot spots (D_10cc_) and the skin volume that receives 52.5 Gy (V_52.5Gy_) are prognostic factors for severe radiation dermatitis.^18^ Therefore, it is essential to use a planning technique that minimizes the skin dose while preserving CTV coverage. The lower skin dose in the MC‐optimized plans could then translate into a lower likelihood of severe radiation dermatitis. Previous studies by Liang et al.^18^ and by Parekh et al.^19^ showed that the risk of moist desquamation is much higher in post‐mastectomy radiation therapy compared with breast conservation settings. Our current study also compared intact breast and CW groups. The CW group had greater improvement in skin sparing with the MC‐optimized plans, as compared with the MC‐renormalized plans. Therefore, despite the planning efficiency trade‐off, we recommend MC optimization due to dosimetric superiority. A hybrid MC‐PB dose algorithm has been proposed recently for proton therapy inverse planning to balance dose accuracy and plan efficiency,^20^ this may potentially offer a better option if adopted by commercial TPSs.

Monte Carlo algorithm simulates particle propagation through materials by randomly sampling the cross sections of interactions. If the physics process modeling is accurate and the number of particle histories is adequate, MC will provide more accurate dose estimation. Therefore, MC dose calculation is considered to be the most accurate method for dose computation in radiation therapy, and this is especially relevant for proton therapy. The dose deposition in proton therapy depends not only on electromagnetic interactions (as in photon therapy) but also on nuclear reactions that are best calculated with MC. For breast cancer IMPT, beams with a range shifter and a relatively large air gap are used to treat the large target volume extended to the patient surface. Accurate modeling of the nuclear halo resulting from the large‐angle scattered particles from nuclear reactions is essential. Proton therapy offers more conformal dose distributions and better OAR sparing for breast cancer, as compared with photon therapy. To ensure these advantages translate into real clinical benefits, accurate dose calculation for breast proton therapy is essential.

In the current study, we have performed a robustness evaluation on the MC‐optimized plans. In the worst case scenario of a 5‐mm setup error and a 3.5% range uncertainty, all MC‐optimized plans reached 95% of CTV covered by at least 90% of the prescription dose, which is our institutional acceptance criteria. In addition, we also chose two extreme cases with the largest and smallest CTV volumes, to perform robustness evaluation on the MC‐renormalized plans. The CTVs’ D_95%_ coverage in the worst case scenario from the MC‐renormalized plans and the MC‐optimized plans were found comparable.

## 5 CONCLUSION

The PB algorithm significantly overestimates the target dose in breast IMPT plans when compared with MC recalculation. The magnitude of the target dose overestimation warrants further investigation to assess clinical significance. The target dose overestimation is higher for CTV CW than for CTV breast. Although MC‐renormalized plans restore the target dose coverage, they lead to significantly increased hot spots in the target and significantly higher skin dose. The MC‐optimized plans are able to provide sufficient target dose coverage, acceptable target hot spots, and good sparing for skin and other OARs. Therefore, utilizing the MC algorithm for both plan optimization and final dose computation in breast IMPT treatment planning is desirable.

## CONFLICTS OF INTEREST

No conflicts of interest.
